# Supplementary figures and images for: The Company of Biologists: celebrating 100 years
Source: Biol Open. 2025 Jan 6;14(1):BIO061842. doi: 10.1242/bio.061842 (PMC11744049; doi:10.1242/bio.061842)

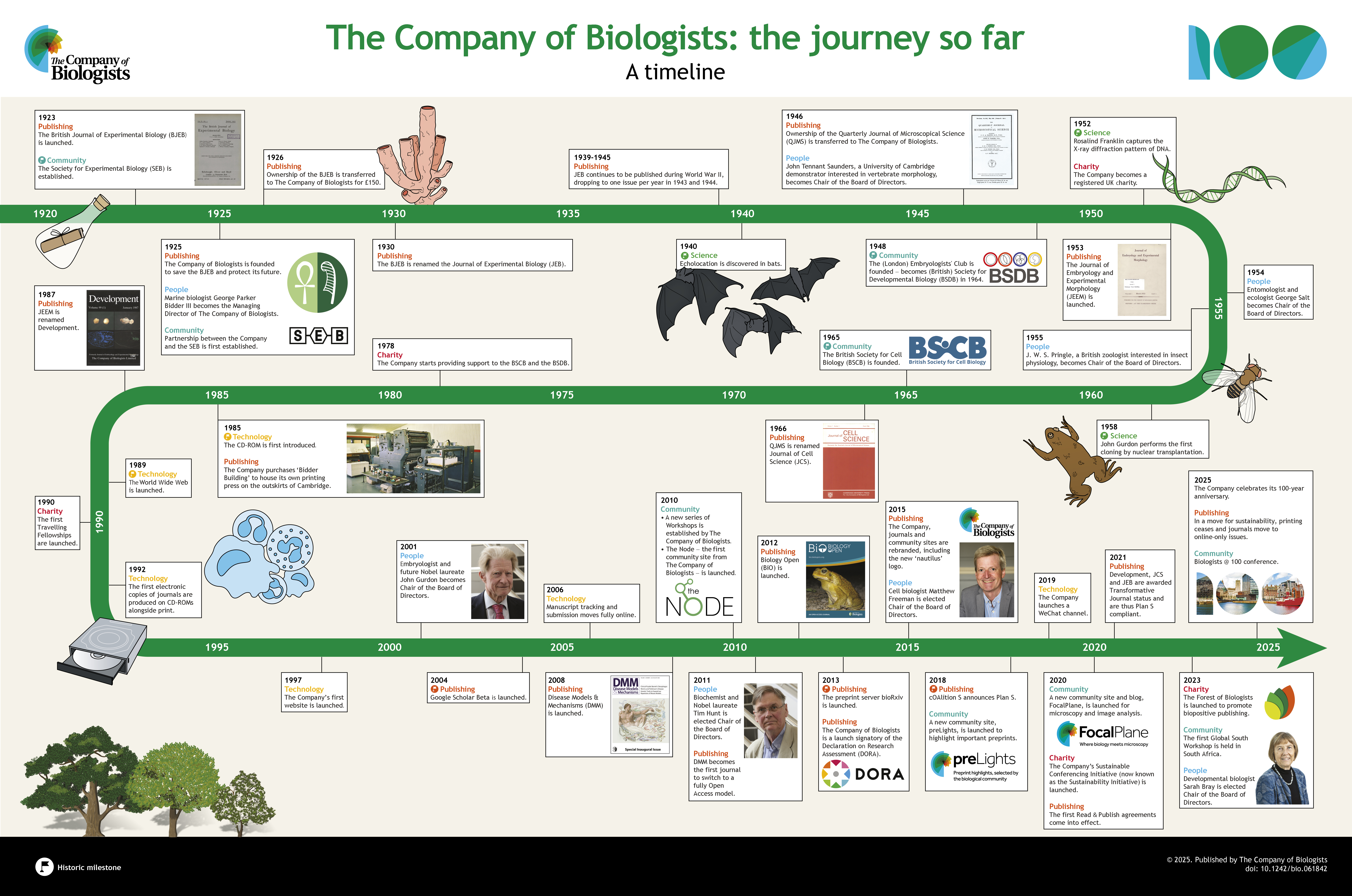

Supplement: Poster [file biolopen-14-061842-s1.jpg]
